# Validation of reference genes for cryopreservation studies with the gorgonian coral endosymbiont *Symbiodinium*

**DOI:** 10.1038/srep39396

**Published:** 2017-01-09

**Authors:** Gabriella Chong, Fu-Wen Kuo, Sujune Tsai, Chiahsin Lin

**Affiliations:** 1 Institute of Marine Biology, National Dong Hwa University, 2 Houwan Road, Checheng, Pingtung, 944, Taiwan; 2 National museum of Marine Biology & Aquarium, 2 Houwan Road, Checheng, Pingtung, 944, Taiwan; 3 Department of Biotechnology, Mingdao University, 369 Wen-Hua Road, Peetow, ChangHua, 52345, Taiwan; 4 Department of Post Modern Agriculture, Mingdao University, 369 Wen-Hua Road, Peetow, Chang Hua, 52345, Taiwan

## Abstract

Quantification by real-time RT-PCR requires a stable internal reference known as a housekeeping gene (HKG) for normalising the mRNA levels of target genes. The present study identified and validated stably expressed HKGs in post-thaw *Symbiodinium* clade G. Six potential HKGs, namely, *pcna, gapdh, 18S rRNA, hsp90, rbcl*, and *ps1*, were analysed using three different algorithms, namely, GeNorm, NormFinder, and BestKeeper. The GeNorm algorithm ranked the candidate genes as follows in the order of decreasing stability: *pcna* and *gapdh* > *ps1* > *18S rRNA* > *hsp90* > *rbcl.* Results obtained using the NormFinder algorithm also showed that *pcna* was the most stable HKG and *ps1* was the second most stable HKG. We found that the candidate HKGs examined in this study showed variable stability with respect to the three algorithms. These results indicated that both *pcna* and *ps1* were suitable for normalising target gene expression determined by performing real-time RT-PCR in cryopreservation studies on *Symbiodinium* clade G. The results of the present study would help future studies to elucidate the effect of cryopreservation on gene expression in dinoflagellates.

Study of gene expression has gradually become an indispensable tool for investigating the effects of various experimental treatments, including cryopreservation. Gene expression is analysed to assess the survivability of cryopreserved samples^1^. Although survivability is the most critical parameter, potential effects of cryopreservation on the genetic material in cells should not be overlooked because alterations in gene expression irreversibly damage cell development^2^. Measurement of gene transcription levels after exposure to a stressor is one type of analyses conducted to determine gene expression. This helps in determining the genetic effect of a particular treatment, such as freezing temperature. Real-time reverse transcription-polymerase chain reaction (real-time RT-PCR) is widely used for quantifying mRNA expression of target genes and allows simultaneous analysis of several genes. Real-time RT-PCR offers the advantages of speed, reliability, sensitivity, ease of operation, and high data throughput compared with other quantification methods such as standard PCR, northern blotting analysis, or ribonuclease protection assay^3^.

Despite its various advantages, quantification of gene expression by performing real-time RT-PCR requires an internal reference for normalising gene expression because gene expression varies based on the quantity of starting material, differences in RNA content between cells, technical variability, and overall transcription efficiency, which hamper effective quantification and relative comparison of a particular gene^1^,^4^. An internal reference is essential to accurately determine the level of gene expression. Stably expressed housekeeping genes (HKGs) are commonly used to normalise gene expression. Typically, HKGs maintain basic cellular functions and are ubiquitously expressed in all cell types regardless of cellular conditions^5^,^6^. However, studies have shown that expression of some of the most commonly used HKGs such as glyceraldehyde-3-phosphate dehydrogenase (*gapdh*), 18S ribosomal RNA (*18S rRNA*), and β-actin is unstable under different treatment conditions and in different cell types^7^,^8^,^9^. Therefore, it is important to validate the expression levels of potential HKGs before using them for normalising target gene expression.

Despite the increasing interest in elucidating the genetic effect of environmental stress on *Symbiodinium* sp. and its coral hosts, studies on suitable HKGs in these dinoflagellates are limited^10^. Significantly fewer genetic studies have been performed on *Symbiodinium* compared with those on vertebrates, with the majority of studies focusing on the effect of temperature elevation^11^,^12^,^13^, light intensity^14^,^15^, and endosymbiotic relationship between *Symbiodinium* sp. and host^12^,^16^. Moreover, use of HKGs in these studies is inconsistent largely because of the nature of these studies and the use of different *Symbiodinium* clades.

In the present study, we identified suitable HKG(s) to normalise gene expression in post-thawed *Symbiodinium* clade G. Real-time RT-PCR was performed to monitor the expression levels of different HKG candidates. Results were analysed using three statistical algorithms, namely, GeNorm, BestKeeper, and NormFinder, to determine the most stably expressed HKG for determining the effects of cryopreservation on target gene expression in *Symbiodinium* clade *G*.

## Results

### Specificity

**Figure 1:**
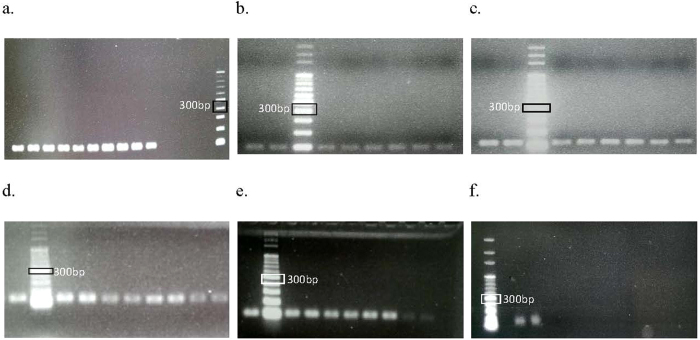
(**a**–**c**) An example of band specificity examined in all sample groups in 1.5% agarose gel by using the specific primers. (**a**) *18S rRNA* (100 bp), (**b**) *gapdh* (96 bp), (**c**) *pcna* (113 bp), (**d**) *rbcl* (126 bp), (**e**) *hsp90* (101 bp), and (**f**) *ps1*. Each band corresponded to the amplicon size of the primer.

**Figure 2:**
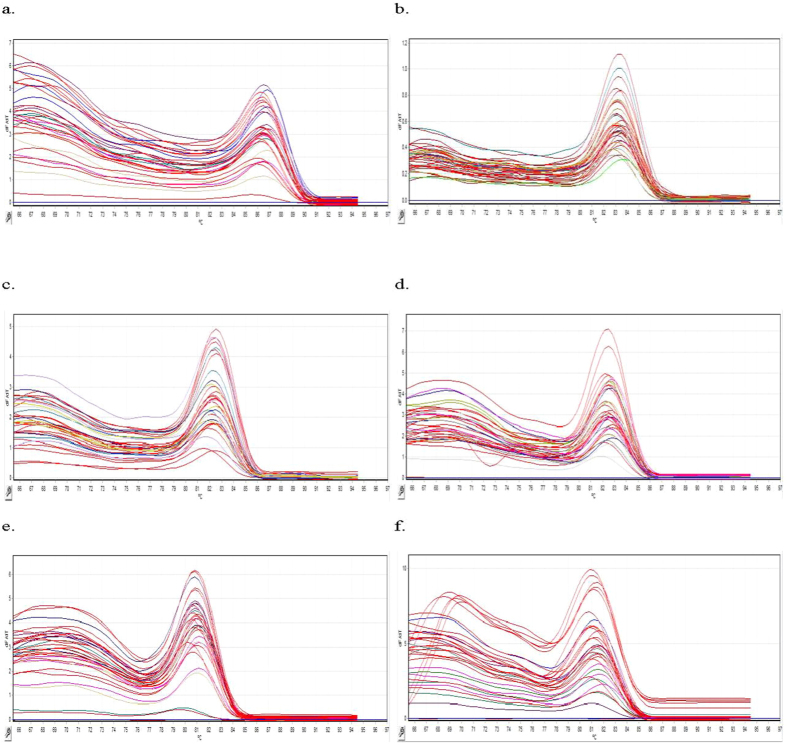
Melting curve of all the candidate HKGs. (**a**) *rbcl*, (**b**) *gapdh*, (**c**) *pcna*, (**d**) *ps1*, (**e**) *18S rRNA*, and (**f**) *hsp90*. Change in fluorescence/change in temperature (−ΔF/ΔT) was plotted against temperature.

Conventional PCR and real-time RT-PCR dissociation were performed to ensure primer specificity. In addition, melting curve analysis was performed after each real-time RT-PCR to ensure primer specificity. Primer specificity was indicated based on the occurrence of only one peak in the melting curve. Gel electrophoresis was performed to verify primer specificity. For conventional PCR, primer specificity was determined using a single band (indicating a single specific product) that migrated the same distance as the expected amplicons relative to a DNA ladder (Fig. 1). All potential HKG primer sets were specific toward *Symbiodinium* sp. samples in control and various post-thawed treatment groups (Fig. 2).

### Analysis of standard curves

Standard curves were plotted using threshold cycle (Ct) values of different standard dilutions against the starting concentration of standard material in the reaction. Standard curve analysis is important to evaluate the performance of a primer. Efficiency, correlation coefficient, and slope of each standard curve were examined before validating the candidate HKGs. For best amplification results, the efficiency and slope of the standard curve should be 100% and −3.32, respectively. In practice, ranges of 90–110% and −3.58 to −3.10 are considered optimum^17^. The efficiency of HKG primers in the present study ranged from 101% for *gapdh* and *18S rRNA* to 112% for proliferating cell nuclear antigen (*pcna*), which slightly exceeded the maximum acceptable range. Slopes ranged from −3.062 for *pcna* to −3.302 for *18S rRNA*. The correlation coefficient measures how well the data fit the standard curve model. Correlation coefficient of ~1.0 indicates better amplification. Correlation coefficients of all the potential HKGs were at least 0.96.

### Ct data

Ct values can be used to determine the approximate transcript abundance of each gene. However, transcript abundance also depends on the efficiency of real-time RT-PCR and variation among sample replicates. Ct values were based on efficiency that was autoselected and was found to be the most optimum by an algorithm of Rotor-gene Q series software. Mean Ct values suggested that rubisco (*rbcl*) was the most abundantly expressed potential HKG, with the lowest Ct value of 23.97 ± 4.48, followed by *18S rRNA*, with a Ct value of 25.11 ± 4.64. Photosystem I, subunit III (*ps1*) was the least expressed candidate gene, with the highest average Ct value of 30.12 ± 2.79 (Table 1).

### Stability of HKG expression

**Figure 3:**
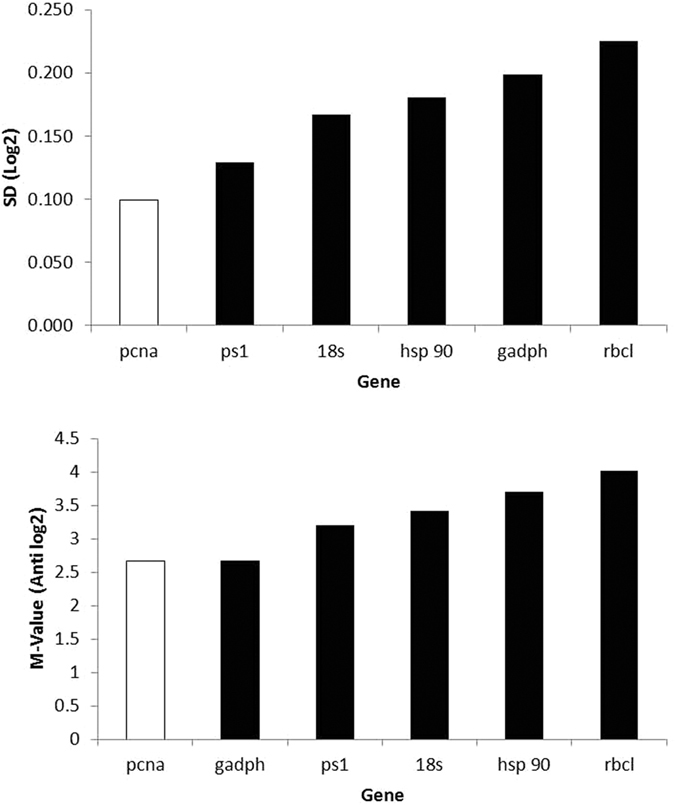
Stability of the candidate HKGs was evaluated using the NormFinder (top) and GeNorm (bottom) algorithms with the GenEx software. Stable genes are indicated by the red bar.

Ct values of each candidate HKG were used to evaluate the stability of gene expression by using the three algorithms GeNorm and NormFinder, and BestKeeper (Fig. 3). Expression of *pcna* was found to be the most stable among the six potential HKGs by both GeNorm (lowest M = 2.66) and NormFinder (stability value = 0.099) algorithms but was ranked at fourth in Bestkeeper (SD ± CP = 3.73) (Table 2). Expression of *gapdh*, a commonly used HKG in many studies also had similar M value with *pcna* by the GeNorm (M = 2.66) but was ranked fifth and the least by both Normfinder (SD = 0.198) and BestKeeper (SD ± CP = 4.35) respectively. *ps1* was ranked second by both NormFinder (SD = 0.129) and BestKeeper (SD ± CP = 2.07) but was placed in the third by the GeNorm (M = 3.2). Evaluation of *hsp90* have showed extremities; it was found to be the most stable (SD ± CP = 1.24) by the BestKeeper but was placed in the fourth and fifth place by both Normfinder and GeNorm with SD = 0.181 and M value of 3.70 respectively. The remaining two genes, *rbcl* and *18sRNA* had similar rankings by these three softwares. *Rbcl* was ranked sixth by both GeNorm (M = 4.01) and NormFinder (0.225) and was second least unstable by the BestKeeper (SD ± CP = 4.27). *18sRNA* was in the third by both NormFinder (SD = 0.167) and BestKeeper (SD ± CP = 3.28) but was ranked fourth by the GeNorm (M = 3.42). Thus the GeNorm algorithm ranked the candidate HKGs in the following order of decreasing stability: *pcna* and *gapdh* > *ps1* > *18S rRNA* > *hsp90* > *rbcl*. The NormFinder algorithm ranked the candidate HKGs in the following order of decreasing stability: *pcna* > *ps1* > *18S rRNA* > *hsp90* > *gapdh* > *rbcl*. And finally, the BestKeeper algorithm ranked the six candidate HKGs in the following order of decreasing stability: *hsp90* > *ps1* > *18S rRNA* > *pcna* > *rbcl* > *gapdh* (Table 2).

## Discussion

The present study identified a stable HKG for normalising gene expression of *Symbiodinium* sp. in cryopreservation experiments. Fewer studies have been performed on HKGs of *Symbiodinium* than those on HKGs of mammals and plants because genome sequences of large number of *Symbiodinium* clades are unavailable. Genome sequences are only available for clades A, B, C, and D because these are common *Symbiodinium* clades present in hard and soft corals, which have been frequently used as test models in thermal- and light-related studies in recent years^13^,^18^,^19^,^20^. Most gene expression studies on *Symbiodinium* sp. involving thermal and light manipulation are conducted using real-time RT-PCR^13^,^18^,^20^,^21^ because of its speed, accuracy, and precision.

In real-time RT-PCR, specificity was evaluated by performing a melting curve analysis (also called dissociation curve analysis) after each amplification. High temperature was used to dissociate dsDNA molecules that associated with fluorescent molecules (e.g., SYBR Green), and the change in fluorescence was determined. Because the dissociation of dsDNA is affected by its length and GC content, different PCR products may have different dissociation characteristics. Specificity can be determined using the melting temperature of the PCR product. A single peak in the −ΔF/ΔT graph denotes a single PCR product (good primer specificity), and two peaks on the melting curve indicate nonspecificity of the primer towards target gene. Primer–dimers may contribute to additional peaks in the melting curve and are identified by lower melting temperatures compared with those of target amplicons. All melting graphs shown here had similar patterns of high −ΔF/ΔT at low temperatures before peaking at expected temperatures. Similar melting curves were observed in a transcriptomic study on human osteoblast genes^22^ where specificity was confirmed by performing gel electrophoresis of PCR products. In the present study, specificity of HKG primers was verified by performing gel electrophoresis, which produced a single band that corresponded to target amplicon size. All HKG primers were found to be specific for our samples. However, all these primers were initially designed for *Symbiodinium* clade C.

Although Ct value data is insignificant in the absence of corresponding efficiency, slope, and correlation parameters, these data can provide a general idea of the relative abundance of gene expression^1^. There is no optimum range for Ct values. However, a range of 20–30 provides reliable and reproducible results. A Ct value of <20 indicates high gene expression, that of >30 indicates low gene expression, and that of >35 indicates no expression^23^. The mean Ct values of the HKG candidates in the present study were within the range of 20–30, except for the Ct value of *ps1*. The Ct value of *ps1* was 30.12 ± 2.79, indicating that its expression was low. High expression (Ct value, <20) does not necessarily indicate that an HKG can be used for normalising target gene expression and could provide erroneous results for genes with low expression^24^. Results of the present study indicated that *rbcl* was the most highly expressed gene, followed by *18S rRNA*. Expression of *rbcl* and its protein in *Symbiodinium* sp. is affected by photoperiod because photosynthesis increases *rbcl* expression^13^. Furthermore, many studies have indicated that *18S rRNA* is one of the most highly expressed genes^24^,^25^. However, expression of *18S rRNA* in the present study was in the range of 20–30. A previous study suggested that use of *18S rRNA* as an HKG will require additional dilution for appropriate normalisation^26^.

A stable HKG is imperative for any study on gene expression at the transcriptional level regardless of the method of analysis because an unstable gene would result in incorrect normalisation of target genes^1^,^27^. In real-time RT-PCR, HKG is used as a normalising standard because of experimental and technical variables that may lead to inconsistent outcomes. It is essential that all HKGs used in any gene expression study, including the most commonly used HKGs such as *gapdh*^7^ and β-actin^28^ that are regulated under different condition, be validated. Stable expression of each HKG in the present study was evaluated using three commonly used algorithms. GeNorm^4^, NormFinder^29^, and BestKeeper^30^ use different algorithms to determine the stability of HKGs. Of the three algorithms, BestKeeper uses raw Ct values, whereas GeNorm and NormFinder use normalised and log-transformed normalised Ct values, respectively. However, because both GeNorm and NormFinder were incorporated in the GenEx software, Ct values were directly entered into the software, and calculations were performed using the GeNorm and NormFinder algorithms.

Previous studies indicate that *pcna, gapdh, 18S rRNA, hsp90*, and *rbcl* have been selected as potential reference genes for determining gene expression in different tissues from various species, including plants^15^,^31^, goat^32^, and fish^33^. In the present study, the three algorithms provided slightly different stability rankings, which was not unexpected because these algorithms use different approaches.

The protein encoded by *pcna* functions as a DNA clamp during DNA replication and facilitates DNA polymerase by increasing the processivity of leading strand synthesis^34^. *pcna* is highly conserved in many species and therefore is a suitable HKG for PCR analysis of gene expression in many organisms, including bacteria^35^. *pcna* is regulated at the post-translational level in dinoflagellate *Pfiesteria piscicida*. At the transcriptional level, *pcna* mRNA is stable and its expression is not affected by different growth phases of cells^36^. However, this is not observed in other dinoflagellates^37^. Although we used different stress treatments in the present study, our results were consistent with those of Boldt *et al*.^20^ who confirmed that *pcna* and β-actin are the most stable genes for analysing gene expression after light manipulation. These two genes have been used as HKGs in thermal stress studies^21^,^38^.

Both *gapdh* and *18S rRNA* are commonly used as HKGs to determine gene expression in different tissues, including fish^7^,^39^, plants^40^,^41^, and human tissue^42^. In the present study, only GeNorm ranked *gapdh* as the most stable however, *gapdh* was ranked as the fifth by the NormFinder and the least stable by the BestKeeper. In plants, *gapdh* is involved in the glycolysis pathway. Translation of the enzyme from *gapdh* mRNA is regulated by diel cycle in dinoflagellate *Gonyaulax polyedra.* However, transcription of the gene is constant throughout the diel cycle^43^. Although *gapdh* was found to be stable at the transcriptional level in both the study by Fagan *et al*.^43^ and in the present study, it was not evaluated for *Symbiodinium* clade C by Boldt *et al*.^20^ because they felt that *gapdh* showed >50% similarity in the region of overlap with the cytosolic isoform. Rosic *et al*.^10^ showed that *gapdh* was an unsuitable HKG for normalising gene expression in thermal and light studies. Nevertheless, *gapdh* is the most stable HKG in ice alga *Chlamydomonas* in freezing acclimation studies^44^, which is consistent with the results of the present study, indicating the stability of *gapdh* mRNA during low-temperature treatment. Moreover, *gapdh* was found to be a suitable HKG for normalising gene expression in red alga *Porphyra yezoensis* at various life stages^24^.

Compared with *gapdh, 18S rRNA* was ranked nearly the same by all three algorithms. Both NormFinder and BestKeeper ranked *18S rRNA* as the third most stable HKG, whereas GeNorm ranked it as the fourth most stable HKG. Protein encoded by *18S rRNA* is a part of the small eukaryotic ribosomal subunit (40 S). Because 18S rRNA is the structural RNA of the small component of eukaryotic cytoplasmic ribosomes, it is one of the most basic components in all eukaryotic cells. Because of the slow evolutionary rate of *18S rRNA*, it is one of the most frequently used genes in phylogenetic studies^45^. The suitability of *18S rRNA* as an HKG varies. Despite its high stability in human tissues^46^, fish^7^, and some plants^47^, many studies have shown that *18S rRNA* is an unsuitable HKG in different plants^25^,^48^,^49^ and *Symbiodinium* clade C^20^. Unsuitability of *18S rRNA* stems from the high expression of ribosomal RNA compared with that of functional mRNA, which may result in erroneous normalisation. However, *18S rRNA* was used as an HKG by Mayfield *et al*.^13^ to study *rbcl* expression in *Symbiodinium* because *18S rRNA* expression is stable in this species.

Evaluation of *rbcl* by the three algorithms yielded similar results. Ribulose-1,5-bisphosphate carboxylase/oxygenase encoded by *rbcl* is one of the main enzymes involved in carbon fixation and is widely found in photosynthetic organisms^50^. Despite its important role in carbon fixation, the actual function of the enzyme in dinoflagellates is relatively unknown because of its short half-life^51^. Nevertheless, *rbcl* was recently found to be a suitable HKG for normalising gene expression in tea plants *Camellia sinensis* and *C. assamica*^52^. However, in *Symbiodinium, rbcl* is rarely used as an HKG because its transcription is highly regulated by light^13^ and temperature^15^. Regulated expression of *rbcl* was consistent with the results provided by the three algorithms, which showed that *rbcl* was an unsuitable HKG.

An interesting finding to the present study was the stability of *hsp90* and *ps1* in Bestkeeper. *hsp90* was showed to be the most stable and *ps1* was ranked the second by the Bestkeeper. Protein encoded by *hsp90* functions as a chaperone that assists in protein folding and protects proteins against heat stress^53^. Heat stress increases the transcription of *hsp90*, indicating that *hsp90* is regulated by temperature stress thus was originally deduced to be unsuitable for temperature related experiments. And although *hsp90* is a suitable HKG for normalising gene expression in different tissues of various species, its use as an HKG in studies on *Symbiodinium* is uncommon. However, it has been used as a target gene in most gene expression studies on *Symbiodinium* that have examined the effect of thermal and light stress^10^,^18^,^21^.

In addition to hsp90, stability of *ps1* was also notably stable in low temperature treatment. The protein encoded by *ps1* functions as subunit III of photosystem I, which is responsible for the efficiency of electron transfer from plastocyanin to cytochrome c553 in algae. This plastocyanin-docking protein contributes to the specific association of plastocyanin with photosystem I and thus plays an important role in photon transfer for photosynthesis^15^,^54^. Several studies have used *ps1* as a target gene. However, its expression was also unexpectedly stable when analysed using NormFinder, which ranked it as the second most stable gene thus indicating the gene stability is treatment dependent and may also be suitable for low temperature experiments. Before selecting the six candidate HKGs in this study, we conducted a preliminary experiment to determine suitable genes and their corresponding primers. These genes included three of the four HKGs validated by Rosic *et al*.^18^, namely, *tub, rp-s4*, and *sam*. These genes were found to be unsuitable because of their nonspecificity. Their band sizes in agarose gels did not correspond to the expected amplicon sizes (results not shown). Other genes, including those encoding major heat shock proteins 40 and 70^18^, D1 protein in photosystem II (*psbA*), P700 protein in photosystem I (*psaA*)^38^, and β-actin, were eliminated because of a similar outcome and because some of these genes were not expressed during conventional PCR.

GeNorm and Normfinder are two common software programs for identifying the most suitable housekeeping gene (HKG) for a particular study, and they tend to produce similar results (but not always) in terms of selecting the most stably expressed gene. The ranking algorithm of NormFinder is generally preferred over that of GeNorm because the latter uses a pairwise correlation calculation that has been found to lack robustness^55^. Given this, although the *pcna* M-value was outside the minimal accepted range for “stable” gene expression classification, NormFinder determined its expression to be stable.

In conclusion, we found that the candidate HKGs examined in this study showed variable stability with respect to the three algorithms. We ultimately chose *pcna* to be the most suitable reference gene for low temperature experiments on *Symbiodinium*, despite GeNorm not supporting such stability. *ps1* (the second most stable HKG) may also be a suitable HKG for low temperature studies of *Symbiodinium*. To our knowledge, this is the first study to validate HKGs for normalizing gene expression data from *Symbiodinium* sp. populations used in cryopreservation experiments and will drive future research on the sub-cellular response of dinoflagellates to sub-freezing temperatures.

## Methods

### Coral collection

*Junceella fragilis* was collected from the Bay of Houwan (21°56′N, 120°56′E), Ping Tung County, Taiwan, in 2014 and 2015. Healthy gorgonian corals were randomly selected and were immediately attached to a rock substrate. The corals were then tagged and were transferred to fresh seawater in an upright position. The corals were kept in tanks with natural seawater throughout the experiments^56^,^57^,^58^. Collection of corals was permitted by the Kenting National Park Management Office (103.0528.1632 and 104.0326.0813).

### Extraction and treatment of dinoflagellates

Dinoflagellates were cryopreserved using an optimised freezing protocol according to a previously described protocol on dinoflagellate extraction and washing^59^. Dinoflagellate samples were incubated with a CPA (1.0 M methanol and 0.4 M sucrose) in straws for 30 min and were placed 5-cm above liquid nitrogen (LN_2_) for 30 min. The samples were then immersed in LN_2_ for at least 10 min before thawing at 37 °C for 10 s in a hot water bath. Viability was assessed by performing ATP viability assay. Only post-thaw dinoflagellate samples with ATP concentrations greater than 40% of those in the control group (without cryopreservation) were cultured for subsequent molecular studies.

### Experimental design for molecular studies

Post-thaw dinoflagellates were cultured for 1, 2, 4, or 8 days to examine differences in gene expression. The control group included freshly isolated dinoflagellates. Freshly isolated and post-thaw dinoflagellates from each time point were used to obtain three biological replicates, each of which was analysed three times. At the end of each culturing period, total RNA was extracted as described in the section below. Six genes (*pcna, gapdh, 18S rRNA, hsp90, rbcl*, and *ps1*) were selected as candidate HKGs (Table 3). Primers were purchased from GMbiolab (Taiwan) and were synthesised by Bio Basic Inc. (Canada). The primers were dissolved in PCR-grade water to obtain a 100 μM stock solution and were diluted to 20 fold by adding 190 μL PCR-grade water. Next, 5 μL each of forward and reverse primers were used for performing real-time RT-PCR.

### RNA extraction

Total RNA was extracted using TRIzol (Roche, Germany), according to the manufacturer’s protocol for plant tissue with some modifications. Algae (either fresh [control] or cultured [treatment]) were collected and were centrifuged at 1000 × *g* for 3 min. Supernatants were discarded, and 2 mL TRIzol was added to the algal pellet and was mixed by pipetting. Next, the TRIzol/algae mixture was transferred to a mortar and was ground continuously in LN_2_ for 45 min into a powdery texture. Chloroform (Sigma, USA) at a ratio of 200 μL for 1 mL TRIzol used was added and was evenly mixed by pipetting. Homogenate obtained was transferred to a 1.5-mL microcentrifuge tube and was mixed by gently flipping and shaking the tube for 30 s. The samples were incubated on ice for 5 min and were centrifuged at 12000 × *g* for 15 min at 4 °C. After centrifugation, each sample showed three liquid layers. Only the upper aqueous layer was collected without disturbing the interphase and the lower phase. The aqueous layer was transferred into a new 1.5-mL microcentrifuge tube, and TRIzol and chloroform were added to the tube at ratios of 1:1 and 1:5, respectively, of the supernatant volume. The solution was mixed by gentle flipping and shaking, was incubated on ice for 5 min, and was centrifuged (12000 × *g* and 4 °C for 15 min) for separating the second phase. The upper aqueous layer was collected and was transferred into a new microcentrifuge tube. An equal volume of isopropanol (Merck, Germany) was added, and the solution was evenly mixed by gently flipping and shaking the tube. The solution was incubated overnight at −20 °C in a refrigerator to allow RNA precipitation. After incubation, the solution was centrifuged at 12000 × *g* and 4 °C for 10 min. Supernatant obtained was discarded, and 1 mL 95% ethanol (Nihon Shiyaku, Taiwan) was added to wash to resuspend the RNA pellet, followed by centrifugation at 12000 × *g* and 4 °C for 6 min. The washing step was repeated twice to clear residual phenol. Ethanol residue was removed by evaporation in a centrifugal evaporator (CentriVap; Labconco, USA). Finally, 16 μL DEPC-treated distilled water was added to dissolve the RNA pellet. RNA quality and quantity were assessed using SSP-3000 Nanodrop spectrophotometer (Infinigen Biotech), and RNA integrity was confirmed by performing gel electrophoresis on 1% agarose in TAE buffer (both from UniRegion Bio-tech). The RNA obtained was reverse transcribed to cDNA for long-term storage, as described below.

### cDNA synthesis

After quantifying total RNA content, 1 μg total RNA was added to each reverse transcription reaction containing TaKaRa Primescript Master Mix. Appropriate volume of the RNA template was mixed with 2 μL MasterMix and PCR-grade water to obtain a final volume of 10 μL. PCR protocol used was as follows: 37 °C for 30 min, 85 °C for 5 s, and finally held at 4 °C. PCR products obtained were stored at −20 °C.

### Standards for real-time RT-PCR

HKG standards were generated by performing conventional PCR with Hotstart Mix (Kapa Biosystems, USA), according to the manufacturer’s protocol^60^. Briefly, 12.5 μL Hotstart Mix was added in a microcentrifuge tube containing 2 μL cDNA template and 2.5 μL of each primer. PCR-grade water was added until the total volume of the reaction mixture reached 25 μL. PCR protocol used was as follows: initial denaturation at 95 °C for 5 min; 40 cycles of denaturation at 95 °C for 30 s, annealing at 60 °C for 30 s, and extension at 72 °C for 10 s; and final extension at 4 °C for 5 min. PCR products obtained were resolved by performing gel electrophoresis on a 1.5% agarose gel in TAE buffer. Bands containing cDNA of interest were excised using a surgical blade atop a blue-light LED epi-illuminator (SMOBIO, Taiwan) and were extracted using AxyPrep DNA Gel Extraction Kit (USA), according to the manufacturer’s protocol. The gel was dried using a paper towel and was minced. The pulp was transferred into a 1.5-mL microcentrifuge tube and was centrifuged using a tabletop centrifuge. Next, gel solubilisation buffer at three times the volume of the gel pulp was added to the tube. The samples were incubated in 75 °C water bath and were vortexed intermittently to completely dissolve the gel. Binding buffer at one-half of the volume of the gel solubilisation buffer used was added to the dissolved gel along with 100 μL propanol. The samples were evenly mixed and were transferred into a new microcentrifuge tube with filters and were centrifuged at 12000 × *g* for 1 min. The filtrate was discarded, 500 μL wash buffer was added to the tube, and the samples were centrifuged for 30 s at the same speed. The filtrate was discarded, 700 μL desalting buffer was added to the tube, and the samples were centrifuged for 30 s. This step was repeated by performing centrifugation for 1 min. Final centrifugation was performed without adding any buffer. The filter column was transferred to a new microcentrifuge tube, and cDNA was eluted using 20 μL DEPC-treated water to the centre of the membrane for 1 min, followed by centrifugation at 12000 × *g* for 1 min. Concentration of the cleaned and eluted PCR product was assessed using Nanodrop spectrophotometer, and the PCR product was diluted to 2 ng/μL.

### Conventional PCR

Before performing real-time RT-PCR, conventional PCR was performed on all samples to determine the suitability of the primers for dinoflagellate samples. Conventional PCR was conducted using KAPA2G Fast HotStart ReadyMix (Kapa Biosystems, USA), according to the manufacturer’s protocol. PCR-grade water was added to 12.5 μL ReadyMix, 2 μL specific primers, and 2 μL template cDNA to make up the final volume to 25 μL. PCR was performed using the protocol for generating standards for real-time RT-PCR. Gel electrophoresis was performed by loading the final PCR products onto a 1.5% agarose gel in TAE buffer, and the gel was viewed under UV light. Primers that were specific toward the candidate genes yielded bands of expected sizes (Table 3).

### Real-time RT-PCR quantification

Real-time RT-PCR was performed using cDNA diluted to 100 fold, 10 μL SYBR FAST qPCR Master Mix (Kapa Biosystems, USA), and 2 μL 20 μM specific primers (Table 3) in a 20-μL reaction mixture. Real-time RT-PCR was performed to quantify the candidate HKGs and to assess their stability of expression across all the samples. Real-time RT-PCR was performed using Rotor-Gene Q real-time RT-PCR system (Qiagen, Germany). All the samples were examined in triplicate, and a no-template control containing deionised water without any cDNA template was used for all the reactions. Standards for each primer were prepared for all the reactions by serially diluting the cDNA to 10 fold to optimise Ct values and to calculate gene concentration. Conditions for real-time RT-PCR were as follows: one cycle at 95 °C for 5 min, followed by 40 cycles of 98 °C for 10 s and 60 °C for 20 s. Real-time dissociation curve was plotted at the end of every reaction to verify primer specificity and to determine the presence of nonspecific PCR products.

### Statistical analysis

Three biological samples were prepared for this experiment, and technical triplicates were performed for each set of biological samples. Before performing stability evaluations, the threshold for gene amplification was established to determine the abundance of gene expression in the form of a raw Ct value. The level of gene expression was correlated with the number of cycles used to amplify the gene to reach the threshold. A low Ct value indicated high gene expression, and a high Ct value indicated low gene expression. A standard curve was constructed for each reaction. Parameters of the graph such as correlation coefficient (R^2^), slope of the curve (M), and efficiency (E) fell within an optimum range before evaluating gene expression. The stability of the candidate HKGs was analysed using the algorithms GeNorm, NormFinder, and BestKeeper. Analyses by using both the GeNorm and NormFinder algorithms were performed with the GenEx software (ver. 6), and analysis by using the BestKeeper algorithm was performed using an original Microsoft Excel-based software.

## Additional Information

**How to cite this article**: Chong, G. *et al*. Validation of reference genes for cryopreservation studies with the gorgonian coral endosymbiont *Symbiodinium. Sci. Rep.* **7**, 39396; doi: 10.1038/srep39396 (2017).

**Publisher's note:** Springer Nature remains neutral with regard to jurisdictional claims in published maps and institutional affiliations.

## Figures and Tables

**Table 1:** Ct values for each candidate HKG.

Gene	Mean ± st dev
*rbcl*	23.97 ± 4.48
*gapdh*	26.83 ± 5.75
*pcna*	27.61 ± 4.56
*hsp90*	27.97 ± 1.66
*18 S rRNA*	25.11 ± 4.64
*ps1*	30.12 ± 2.79

**Table 2:** The ranking of the potential HKGs according to GeNorm, NormFinder and BestKeeper.

Candidate HKGs	GeNorm	NormFinder	BestKeeper
*pcna*	1	1	4
*gapdh*	1	5	6
*18S rRNA*	4	3	3
*rbcl*	6	6	5
*ps1*	3	2	2
*hsp90*	5	4	1

**Table 3:** Genes and their respective primer sequence used in this study.

Gene	Full gene Name	Primer Sequence (5′-3′)	Amplicon size (bp)	Reference
*pcna*	Proliferating cell nuclear antigen	F: GAGTTTCAGAAGATTTGCCGAGAT	113	Boldt *et al*.^20^
		R: ACATTGCCACTGCCGAGGTC		
*gadph*	Glyceraldehyde-3- phosphate dehydrogenase	F: GCCCTGCGTGTGCCAACCAT	96	Leggat *et al*.^21^
		R: CTTCATCTCAGCGCAAATCTCCTCGTA		
*18S rRNA*	18S ribosomal RNA	F: CAAGCCCGACTTTGCAGA	100	Mayfield *et al*.^13^
		R: CGCACGATTCGTCAAGTTATC		
*hsp90*	Heat shock protein 90	F: GAGGATCTGCCACTGAACATCTC	101	Rosic *et al*.^10^
		R: GCGAACATCTCCAAGCACTTC		
*rbcl*	Rubisco	F: CAGTGAACGTGGAGGACATGT	126	Mayfield *et al*.^13^
		R: AGTAGCACGCCTCACCGAAA		
*ps1*	Photosystem I (subunit III)	F: GTGGAGTTGACATTGACTTGGA	136	Mayfield *et al*.^15^
		R: TGCTGCTTGGTGGTCTTGTA		
